# High-efficiency decomposition of eggshell membrane by a keratinase from *Meiothermus taiwanensis*

**DOI:** 10.1038/s41598-022-18474-4

**Published:** 2022-08-29

**Authors:** Ya-Chu Lien, Shu-Jung Lai, Chai-Yi Lin, Ken-Pei Wong, Matt S. Chang, Shih-Hsiung Wu

**Affiliations:** 1 Institute of Biological Chemistry, Academia Sinica, No. 128, Academia Road, Section 2, Nankang, Taipei, 11529 Taiwan; 2 Graduate Institute of Biomedical Sciences, China Medical University, No. 91, Hsueh-Shih Road, Taichung, 40402 Taiwan; 3 Research Center for Cancer Biology, China Medical University, No. 91, Hsueh-Shih Road, Taichung, 40402 Taiwan; 4 I-MEI FOODS Company Limited, 1 F., No. 31, Sec. 2, Yanping N. Rd., Datong Dist., Taipei City, 10346 Taiwan

**Keywords:** Biological techniques, Biotechnology, Health care

## Abstract

Eggshell membrane (ESM), a plentiful biological waste, consists of collagen-like proteins and glycosaminoglycans (GAGs) such as hyaluronic acid (HA). Here we used a keratinase (oeMtaker)-mediated system to decompose ESM. The best reaction condition was established by incubating the solution containing oeMtaker, sodium sulfite, and ESM with a weight ratio of 1:120:600. ESM enzymatic hydrolysate (ESM-EH) showed a high proportion of essential amino acids and type X collagen peptides with 963–2259 Da molecular weights. The amounts of GAGs and sulfated GAGs in ESM-EH were quantified as 6.4% and 0.7%, respectively. The precipitated polysaccharides with an average molecular weight of 1300–1700 kDa showed an immunomodulatory activity by stimulating pro-inflammatory cytokines (IL-6 and TNF-α) production. In addition, a microorganism-based system was established to hydrolyze ESM by *Meiothermus taiwanensis* WR-220. The amounts of GAGs and sulfated GAGs in the system were quantified as 0.9% and 0.1%, respectively. Based on our pre-pilot tests, the system shows great promise in developing into a low-cost and high-performance process. These results indicate that the keratinase-mediated system could hydrolyze ESM more efficiently and produce more bioactive substances than ever for therapeutical applications and dietary supplements.

## Introduction

From ancient times to the present, eggs have been one of the delicacies of human nutrition. Due to vast market demand, the number of processed egg products is considerable. The global annual production quantities of eggs reached 83 million tons in 2019^1^. However, with the manufacturing of egg products the amount of biological waste, that is produced is large. Prominent among these wastes are eggshells, how to treat and reuse these wastes effectively and in an environment-friendly way has become an important issue. Eggshell membrane (ESM), serving as a scaffold between the egg white and the eggshell, is composed of two layers of water-insoluble fibrous mesh membranes^2^. ESM is composed of 80–90% protein, including a high proportion of type I, V, and X collagen and other multifunctional proteins^3,4^ such as ovotransferrin^5^, lysyl oxidases^6^, lysozymes^7^, and β-N-acetylglucosaminidase^8^. ESM also contains bioactive components including glucosamine, hyaluronic acid (HA), chondroitin sulfate, and dermatan sulfate^9,10^ which have been used in product development due to properties such as moisture retention. The anti-viral and anti-inflammatory characteristics of degraded ESM could help improve joint-related symptoms, smooth skin wrinkles, and accelerate wound healing^11,12^. However, the water-insoluble ESM is extensively crosslinked with disulfide bonds, causing a significant challenge to its decomposition^13,14^.

At present, ESM is decomposed by using strong acid/alkali solutions or incubated at high temperatures under high-pressure conditions. After treatment with these chemical and physical decomposition procedures, the resulting molecules may become non-absorbable or lose their nutritious components for further application^15^. Therefore, the development of ESM enzymatic hydrolysis has attracted attention. Many hydrolytic enzymes, such as papain, trypsin, and pepsin, have been reported to digest ESM and release absorbable nutrient molecules^16^. Unlike these enzymes, keratinase is a broad-spectrum serine protease that belongs to the subtilisin family^17,18^. The enzyme was named for its powerful activity that can hydrolyze keratin, a very robust protein. Consequently, we studied the use of an extracellular thermostable keratinase (Mtaker) from the thermophilic bacteria *Meiothermus taiwanensis* WR-220^19^ in ESM hydrolysis.

In order to treat and reuse ESM effectively by environment-friendly procedures, the keratinase-mediated ESM decomposition system was optimized in this study. The components of the enzymatic hydrolysate (ESM-EH), such as free amines, glycosaminoglycans (GAGs), and sulfated GAGs were quantified. We quantified the bioactive molecules that can be reused based on proteomics analysis and amino acid composition from ESM-EH. The major polysaccharides from ESM-EH were precipitated by ethanol and characterized using NMR, GC-MS, and FTIR. These glycans induced macrophages to produce IL-6 and TNF-α, which possess immunomodulatory activity. In addition, a microorganism-based ESM hydrolysis system was optimized by using ESM as the sole carbon and nitrogen source to culture *M. taiwanensis* WR-220, which dramatically decreases the cost and is easier to scale up in industrial application. The flowchart of full experiments in this report is shown in Supplementary Fig. S1.

## Methods

### The source of ESM

All ESM samples were provided by I-MEI Foods Co., Ltd. (Taiwan) and shelled, cleaned, and dried. Two types were used: the original sheet shape (ESM) and a ground powder (ESMP).

### Optimization of the ESM enzymatic hydrolysis (ESM-EH) conditions by oeMtaker

The purified keratinase heterologous overexpressed in *E. coli* was named oeMtaker. To determine the concentration dependence of oeMtaker on ESM solubilization, a test solution of 30 mg ESMP, 50 μL of 1 M Na_2_SO_3_ in 1 mL keratinase buffer (30 mM HEPES, 250 mM NaCl, pH 8.0) was used. Incubation was carried out under conditions in which the final concentrations were 50 mM Na_2_SO_3_ and 0, 0.1625, 0.3650, 0.650 and 1.300 (μM) oeMtaker (12.3 ± 0.7 U). In other words, the weight ratio of the corresponding ESM is 0:30, 1:6000, 1:3000, 1:600, and 1:300. For sodium sulfite concentration dependence of ESM solubilization, the test solution was 30 mg of ESMP, 0.05 mg of oeMtaker in 1 mL keratinase buffer. Incubation was carried out under conditions that the final concentrations were 0.650 μM oeMtaker and 0, 10, 20, 30, 50, 100 and 200 mM Na_2_SO_3_. That is, the weight ratio of the corresponding ESM was 0:30, 1:23.1, 1:12.0, 1:7.9, 1:4.8, 1:2.4, and 1:1.2. Each sample was subjected to hydrolysis reaction at 55 °C, with 1000 rpm shaking for 3 h. After incubation, each ESM-EH sample was harvested by centrifugation at 17,000 × g, 4 °C for 10 min. The supernatant and remaining ESM were collected for further experiments.

### Keratinase activity determination.

Keratinase activity was determined by the keratin azure method^20^. oeMtaker or another sample (100 μL, 1 mg/mL) was incubated with 4 mg of keratin azure (Sigma, USA) in 1 mL of Tris–HCl buffer (0.1 M, pH 8.0) at 55 °C with 300 rpm orbital shaking for 1 h. The reaction was terminated by adding 55 μL trichloroacetic acid (Sigma, USA). Samples were centrifuged at 17,000 × g for 10 min, and the absorbance of the released azure stain in the supernatant was determined at 595 nm. In enzyme blanks, TCA solutions were added before the reaction. One unit (U) of keratinase was defined as the amount of enzyme that increase of 0.01 in absorbance at 595 nm.

### Decomposition percentage measurement of the keratinase-mediated ESM system

After each reaction, 2 mL of DD water (repeated twice) was added to wash the remaining ESM. Then, the residual ESM was placed in a 55 °C oven to dry. After cooling to room temperature, it was weighed. The decomposition percentage (%) of ESM by the keratinase-mediated ESM decomposition system was calculated using the following formula^20^.

$$\frac{{{\text{Original}}\,\,{\text{ESM}}\,\,{\text{weight}}\,\,\left( {{\text{mg}}} \right) - {\text{Residual}}\,\,{\text{ESM}}\,\,{\text{weight}}\,\left( {{\text{mg}}} \right)}}{{{\text{Original}}\,\,{\text{ESM}}\,\,{\text{weight}}\,\left( {{\text{mg}}} \right)}} \times 100\%$$

### Concentration measurement of free amines in the ESM-EH

The concentration of free amines of the ESM-EH was quantified by the ninhydrin colorimetric method^21^. ESM-EH supernatant (100 μL) was mixed with 50 μL of ninhydrin reagent (Sigma–Aldrich, Inc.). The reaction mixture was incubated at 100 °C for 10 min, and then 75 μL of the sample was loaded into a 96-microwell plate as it was cooling. The reaction mixture was mixed well with 125 μL of 95% ethanol for subsequent absorbance measurement at 570 nm. Glycine (Sigma, USA) was used as a standard.

### Analysis of amino acid composition in the ESM-EH

This analysis was entrusted to Mission Biotech Co., Ltd. (Taiwan). A TRAQ Kit was used to perform amino acid composition and quantitative analysis by Liquid Chromatography Mass Spectrometry (LC-MS). The sample preparation used 0.650 μM oeMtaker with 50 mM Na_2_SO_3_ to hydrolyze the ESM at 55 °C for 3 h. After being hydrolyzed, the upper part of the ESM-EH was filtered by a 0.22 μm PVDF filter membrane and then calibrated according to the original experimental procedure of the TRAQ Kit^22^.

### Analysis of oligopeptides in the ESM-EH

Oligopeptides from ESM-EH were analyzed by Liquid-Chromatography with tandem Mass spectrometry (LC-MS-MS, Thermo LTQ-Orbitrap Velos)^23^. Sample preparation was carried out as follows: first, the ESM-EH was passed through a 3 kDa centrifugal filter column, the liquid in the lower layer of the column (molecular weight less than 3 kDa) was collected; a Pierce^TM^ Graphite Spin Columns Kit (Thermo)^23^ was used to remove impurities and salt, and the sample was freeze-dried and stored at –80 ℃. Next, 0.5 μg of the freeze-dried sample was dissolved in 0.1% formic acid and injected into LC-MS with a scanning range of 450–2000 m/z, and secondary fragmentation (MS/MS) was performed with prominent peaks. Finally, the obtained mass spectrum data were loaded into the MaxQuant (ver. 1.6.6.0)^24^ with *Gallus gallus* total protein database downloaded on 11th Jun 2021 from Uniprot (https://www.uniprot.org)^25^. If the score was more than 40 points, the result was considered credible.

### Extraction and purification of polysaccharides from the ESM-EH

The polysaccharide was precipitated by mixing the ESM-EH supernatant with three volumes of cold absolute alcohol (≥ 99.5%, Sigma–Aldrich, Inc.) and then incubated at 4 °C overnight. After centrifugation at 2,850 xg, 4 °C for 20 min, the pellet was harvested and dissolved with one volume of NaCl (1.5 M). Three additional volumes of ethanol were added, and the solution was centrifuged again^26,27^. The crude extract of polysaccharide pellet was resolved with DD water and then freeze-dried, providing a fraction named ESM-C. For further purification^26,27^, ESM-C (20 mg) was dissolved in 1 mL DD water and purified by size exclusion chromatography using an HW-65 column (TSK-gel, 10 kDa-1000 kDa, 1.6 diameters × 90 cm height) to make three polysaccharide fractions, named ESM-C-A (11.3 mg), ESM-C-B (0.1 mg), and ESM-C–C (0.1 mg) (Fig. S4). The isolated carbohydrate fractions were detected by the phenol–sulfuric acid method (see Supplemental information).

### Monosaccharide composition analysis of ESM-C

The monosaccharide composition analysis was determined by GC–MS^28^. ESM-C (approximately 0.5 mg) was methanolyzed with 0.5 M methanolic/HCl (Sigma–Aldrich, USA) at 84 °C for 16 h. After cooling, a solution containing 500 μL methanol, 50 μL acetic acid, and 10 μL pyridine was added and vortexed for 30 min at room temperature. After evaporation to dryness under a stream of N_2_ gas, the reactants were treated with Sylon HTP (HMDS/TMCS/pyridine, 2:1:10) trimethylsilyation reagent (Supelco, PA) at 85 °C for 30 min. The final trimethylsilyated (TMS) derivatives were dissolved in 400 μL *n*-hexane (GC grade, Supelco, PA, USA) for analysis on a Bruker SCION SQ GC–MS instrument (Bruker, Billerica, USA), which was fitted with a DB-5MS fused silica capillary column (length 30 m, inner diameter 0.25 mm, Agilent J & W Scientific, USA). Sodium hyaluronate, L-fucose, D-glucose, D-galactose, D-mannose, D-glucosamine, D-galactosamine, N-acetyl-D-glucosamine, and N-acetyl-D-galactosamine (all Sigma) were also treated with the above derivatization reactions and used as standards.

### NMR spectroscopic data collection

The crude extract of polysaccharide ESM-C sample (15 mg) and the purifier fraction ESM-C-A sample (5 mg) were each dissolved in 400 μL deuterium oxide (99.9 atom% D, Sigma-Aldrich, Inc.) for data collection. The ^1^H chemical shift spectrum was referred to D_2_O at δ_H_ 4.49 ppm (313 K) as an internal standard. For structural elucidation of ESM-C-A, ^1^H and ^13^C spectra were recorded on Bruker AVII-500 MHz and Bruker AVIII-800 instruments, respectively. In the ^13^C NMR spectra measurement, a relaxation delay and acquisition time of 3.5 and 1.3 s, respectively. And 13312 scans per spectrum were accumulated. A standard spectrum of HA at 300 MHz ^1^H-NMR was used as reported^29^.

### The molecular weight of polysaccharides in ESM-EH

The molecular weight (Mw) of the purified polysaccharides in ESM-EH (ESM-C-A) was determined using size-exclusion chromatography on HPSEC equipped with SEC 1000 Guard Column (Thermo Scientific BioBasic) at 35 ℃ with pure water as the mobile phase (flow = 1.0 mL/min) and a refractive index detector^26^. Standards of dextran (Sigma) with different molecular weights (10 kDa, 70 kDa, 500 kDa, and 2000 kDa) were used for calibration (Fig. S5b).

### Microorganism-based ESM hydrolysis system

The aerobic and thermophilic *M. taiwanensis* WR-220 (ATCC BAA-400) isolated from Wulai Hot Spring in northern Taiwan was used in the ESM hydrolysis system^30^. The strain was routinely cultured in Thermus modified (TM) medium at pH 8.0, 55 °C with 190 rpm orbital shaking and usually sub-cultured twice before start-up ESM fermentation. The submerged fermentation process was then initiated by adding the ESM (3 g) or ESMP (3 g) as the sole carbon and nitrogen sources to the basal media (100 mL) in sterilized conical flasks (250 mL), respectively. The cultures were incubated at pH 8.0, 55 °C, and 190 rpm. After 24, 48, and 144 h incubation, the samples (2 mL) were harvested for cell density determination (OD600), free amine concentration detection, oligopeptide concentration detection (Bradford assay), and carbohydrate concentration detection (OD490).

### Determination of GAGs and sulfated GAGs concentration

ESM-EH (1 mL) was further purified using Bio-Gel P-6 column chromatography. Fractions that contained the sugar were collected from ESM-EH and samples were obtained after lyophilization. The concentration of GAGs in the hydrolyzate was by the carbazole method^31^. Briefly, the organic components were released after hydrolysis of GAGs by sulfuric acid and then detected with carbazole reagent which can be observed at 530 nm. The reaction mixture contained 80 μL of sample and 400 μL of 0.025 M sodium tetraborate (Sigma) in sulfuric acid. The mixture was incubated at 100 °C for 15 min and then mixed with 0.125% (w/v) of carbazole in absolute ethanol (16 μL) after cooling to room temperature. The mixed solution was incubated for another 15 min at 100 °C. After cooling to room temperature, its absorbance was detected at 530 nm. GAGs were quantified based on the calibration line of sodium hyaluronate (Sigma–Aldrich). The concentration of sulfated GAGs in ESM-EH was determined by the dimethylmethylene blue assay (DMMB)^32^. This method uses dimethylmethylene blue to react with sulfated GAGs (such as chondroitin sulfate, dermatan sulfate, etc.) to change the absorption value of the reagent. After adding 10 μL of sample to 100 μL of DMMB reagent, the absorbance was measured at a specific wavelength of 525 nm within 10 min.

### Inflammatory activity

The purified polysaccharides in ESM-EH (ESM-C-A) (1 mg) was dissolved in 1 mL DD water to prepare a stock solution. Subsequently, the stock solution was passed through a sterile 0.22 μm filter. The murine J774A.1 macrophage cell line was obtained from 308 American Type Culture Collection (Rockville, MD). The cells were cultured in RPMI-1640 medium supplemented with 10% fetal bovine serum (FBS) and maintained at 37 ℃ under a humidified atmosphere of 5% CO_2_ in an incubator^28^. Next, J774A.1 macrophage cells (1 × 10^5^ cells/well) were cultured in a 24-well plate and treated as indicated for 24 h. Released mouse IL-6 and mouse TNF-α were determined by ELISA according to the manufacturer’s instruction (Thermo Fisher Scientific, Rockford, IL). Polymyxin B (PMB, 5 μg/ml) was treated at 37 ℃ for 30 min to confirm the contamination by LPS^33^. Treatment with LPS (1 μg/mL) served as the positive control in this test. Each data point represents the average from at least triplicate detections.

## Results

### Optimization of the keratinase-mediated ESM hydrolysis system

**Figure 1:**
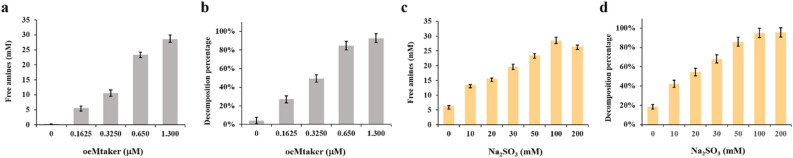
Dose dependent of keratinase (oeMtaker) and sodium sulfite (Na_2_SO_3_) in ESM decomposition assays. The concentration of free amines (**a**) and decomposition percentage (**b**) in the ESM enzymatic hydrolysate (ESM-EH) was each measured at fixed concentration of 50 mM sodium sulfite. The quantified free amine (**c**) and decomposition percentage (**d**) in the ESM-EH when the reaction contained 0.65 μM of oeMtaker. Reactions were incubated at 55 °C for 3 h in a 1000 rpm orbital shaker. All data were obtained and averaged from at least three independent experiments.

Since the water-insoluble ESM with extensive cross-linkages by disulfide bonds is similar to keratin, we hypothesized that our previously discovered broad-spectrum keratinase, oeMtaker, from *M. taiwanensis* WR-220 with high stability in water could also decompose ESM efficiently. With the purpose of optimizing the Mtaker-mediated ESM hydrolysis conditions, we heterologously overexpressed Mtaker (oeMtaker) in *E. coli* and purified it for further experiments. Dose-dependence assays of oeMtaker in ESM decomposition were carried out in the buffer system as described before^19^. The addition of sodium sulfite in each reaction was fixed to 50 mM to determine the ESM decomposition percentage with different doses of oeMtaker. Based on the determination of free amine from ESM-EH at several doses of oeMtaker, reactions containing 0.65 μM oeMtaker were considered to have the highest efficiency in hydrolyzing ESM. The increase in free amine using 1.3 μM oeMtaker was not significantly higher than 0.65 μM oeMtaker (Fig. 1a). Based on the weight calculation of the remaining ESM, the highest ESM decomposition percentage reached 93% and 85% with the addition of 1.3 and 0.65 μM of oeMtaker (Fig. 1b). Therefore, we further determined the dose-dependence of sodium sulfite in ESM decomposition assays using a fixed concentration of 0.65 μM oeMtaker. Among the tested doses of sodium sulfite, the reaction containing 100 mM of sodium sulfite released the highest amount of free amine (28.6 mM, Fig. 1c). Decomposition percentage reached 20% with the addition of oeMtaker but without sodium sulfite to hydrolyze ESM (Fig. 1d). The percentage of decomposition were significantly increased when the sodium sulfite concentrations were increased (Fig. 1d). This finding indicated the higher decomposition efficiency in ESM by oeMtaker with the addition of sodium sulfite. Sodium sulfite has been known to have the ability to reduce chemical crosslinking. Acting as a reducing agent, it will convert the disulfide bond into two sulfonic acid groups, and inhibit free mercaptan groups^34^. According to the calculation of decomposition percentage with several doses of sodium sulfite, there was no significant increase when 100 mM of sodium sulfite was added. Therefore, based on the consideration of cost and hydrolysis efficiency, we determined that reactions containing 50 mM of sodium sulfite and 0.65 μM oeMtaker were the best condition for hydrolyzing ESM. These results indicate an optimal condition for keratinase-mediated ESM decomposition with a 1:120:600 weight ratio of oeMtaker:Na_2_SO_3_:ESM, with more than 85% decomposition percentage of ESM could be obtained within a 3 h reaction.

### Analysis of amino acids composition in ESM-EH

To compare the differences in ESM-EH obtained by treatment with strong acid or with oeMtaker, we analyzed the composition of amino acids in the oeMtaker ESM-EH (Table 1). The ESM of the strong acid method was hydrolyzed with 6 N HCl containing 0.1% (wt/vol) phenol for 24 h at 110 °C in sealed tubes under nitrogen^35^. Each amino acid from ESM-EH was identified and quantified by LC-MS. Due to the limitations in distinguishing Asn/Asp, Gln/Glu, and cysteine/cystine based on samples obtained by strong acid hydrolysis, these three groups were combined to calculate the amino acid ratio. The top-five highest amounts of amino acids from acid hydrolysates or oeMtaker-mediated hydrolysates were Pro/ Glx/Gly/Ser/Asx and Ser/Glx/Cys/Met/Gly, respectively (Table 1). Both treatments showed a higher amount of Glx/Gly/Ser amino acids in hydrolysates, indicating high content of these three kinds of amino acids in ESM. The amounts of methionine obtained from oeMtaker-mediated hydrolysates were higher than those from acid hydrolysates. Methionine is widely used in nutritional supplements and animal feed for application. In the overall amino acid composition, the acid hydrolysis from the literature provided 34.7% of essential amino acids; the keratinase hydrolysis provided 35.4% of essential amino acids that have the potential to develop in various food applications. The amino acid compositions between two treatments, acid hydrolysis and oeMtaker-mediated hydrolysis, identified proline and hydroxyproline from oeMtaker-mediated hydrolysates were significantly lower than acid hydrolysates (Table 1). Proline and hydroxyproline are the major amino acids in collagen, whereas proline is the more frequently released amino acid by the acid hydrolysis method. This reveals that the high-economic-value component in ESM, collagen, was destroyed during acid hydrolysis but not when using the oeMtaker-mediated hydrolysis.

**Table 1:** The composition of amino acids in ESM hydrolysate (ESM-EH).

Individual	Acid hydrolysis^a^	oeMtaker-Na_2_SO_3_
	Average ± SD (%)	
Alanine	4.4 ± 0.3	5.8 ± 1.2
Arginine	5.8 ± 0.4	4.8 ± 1.0
Asx (Asparagine + Aspartic Acid)	8.6 ± 0.4	5.1 ± 0.8
Cys (Cysteine + Cystine)	–	9.4 ± 2.0
Glx (Glutamine + Glutamic Acid)	11.5 ± 0.5	10.1 ± 1.5
Glycine	10.9 ± 0.3	7.9 ± 1.6
Histidine	4.2 ± 0.6	4.5 ± 0.9
Hydroxyproline	1.5 ± 0.5	0.1 ± 0.0
Isoleucine	3.4 ± 0.4	1.7 ± 0.4
Leucine	5.2 ± 0.5	3.7 ± 0.8
Lysine	3.5 ± 0.3	3.3 ± 0.7
Methionine	2.3 ± 1.4	8.3 ± 1.7
Phenylalanine	1.6 ± 0.1	2.7 ± 0.6
Proline	11.8 ± 1.1	0.7 ± 0.1
Serine	9.2 ± 0.2	16.9 ± 3.5
Threonine	6.9 ± 0.2	5.3 ± 1.1
Tryptophan	–	1.4 ± 0.3
Tyrosine	2.0 ± 0.3	3.9 ± 0.8
Valine	7.6 ± 0.2	4.5 ± 0.9

–, Not detected; ^a^Data was averaged from amino acid composition of inner shell membrane and outer shell membrane which decomposed by acid hydrolysis^35^, which used acid hydrolysis method to decompose ESM.

### Identification of oligo-peptides from ESM-EH

We used proteomics methods to identify the amino acid sequence and composition of oligopeptides in ESM-EH. Briefly, the oeMtaker-mediated ESM-EH was centrifuged to remove insoluble components and the supernatant was desalted by using Pierce^TM^ Graphite Spin Columns. Desalted peptides were lyophilized. After analysis by LC-MS-MS, the generated raw data was processed through software and compared with the protein database of *Gallus gallus* in UniProt. According to the score 40 cut-offs, a total of 70 peptides were identified, from which 21 were annotated as peptides from the α1 chain of type X and type XVII collagen (Table 2). Hydroxyproline (Hyp) in collagen is critical in hydrogen bond formation, making the collagen’s structure more stable and firmer^36^. The range of molecular weight from these identified peptides on collagen was 963–2259 Da, which is suitable for application as nutritional supplements^37^. In addition, ESM-EH contains lysine oxidase homolog 2, which can promote collagen cross-linking^38^, and vitellogenin-2, which can promote muscle growth and prevent obesity^39^ (Supplementary Table S1).

**Table 2:** Identified collagen peptides in ESM hydrolysate by LC–MS/MS analysis.

Proteins ID	Peptides sequence	MS/MS m/z	Mass	Score
Collagen α1 (X) chain	GPP*GEP*GEVGIGKP*GPM	812.8872	1622.7610	202
	RGPEGPP*GFP*GP*KGDQGPA	933.4399	1864.8704	170
	RGEQGPP*GPP*GPIGPR	800.9127	1599.8118	166
	GPP*GEP*GEVGIGKP*GP*M	820.8852	1638.7559	166
	GKP*GFGSP*GPQ	530.7560	1059.4985	158
	GLP*GARGPQGPP*GIP*GPA	822.4266	1642.8427	156
	GPPGEP*GEVGIGKP*GPM	804.3881	1606.7661	143
	M(ox)RGEQGPP*GPP*GPIGPR	583.2883	1746.8472	131
	GPIGPP*GMP*GAP*GAK	676.3356	1350.6602	128
	GLP*GSP*GLP*GF	523.7610	1045.5080	119
	GERGLP*GLDGKP*GYP*GEQ	937.9473	1873.8806	119
	GIGKP*GENGLP*GQP*GMK	842.9188	1683.8250	117
	GPP*GEP*GEVGIGKP*GP*M	820.3834	1638.7559	116
	AGIP*GPQGPP*GEP*GEVGIGKP*GPM	754.3673	2259.0842	112
	GIP*GPQGPP*GEP*GEVGIGKPGPM(ox)	1095.5327	2188.0470	111
	PP*GPPGP*IGPR	537.2899	1072.5665	108
	GKP*GYP*GEQ	482.7219	963.4298	104
	AGIP*GPQGP*PGEP*GEVGIGKP*GPM	1131.0480	2259.0842	103
	RGPEGPP*GFP*GPKGDQGPA	925.9432	1848.8755	101
Collagen α1 (XVII) chain	GP*PGPP*GPP*GF	512.7395	1023.4662	191
	GPP*GPP*GPP*GFS	556.2568	1110.4982	93

P*: hydroxyproline.

The data was afforded on the MaxQuant (ver. 1.6.6.0) and *Gallus gallus* total protein database which was downloaded on 11th Jun 2021 from Uniprot (https://www.uniprot.org).

### Identification and characterization of precipitated polysaccharides from ESM-EH

**Figure 2:**
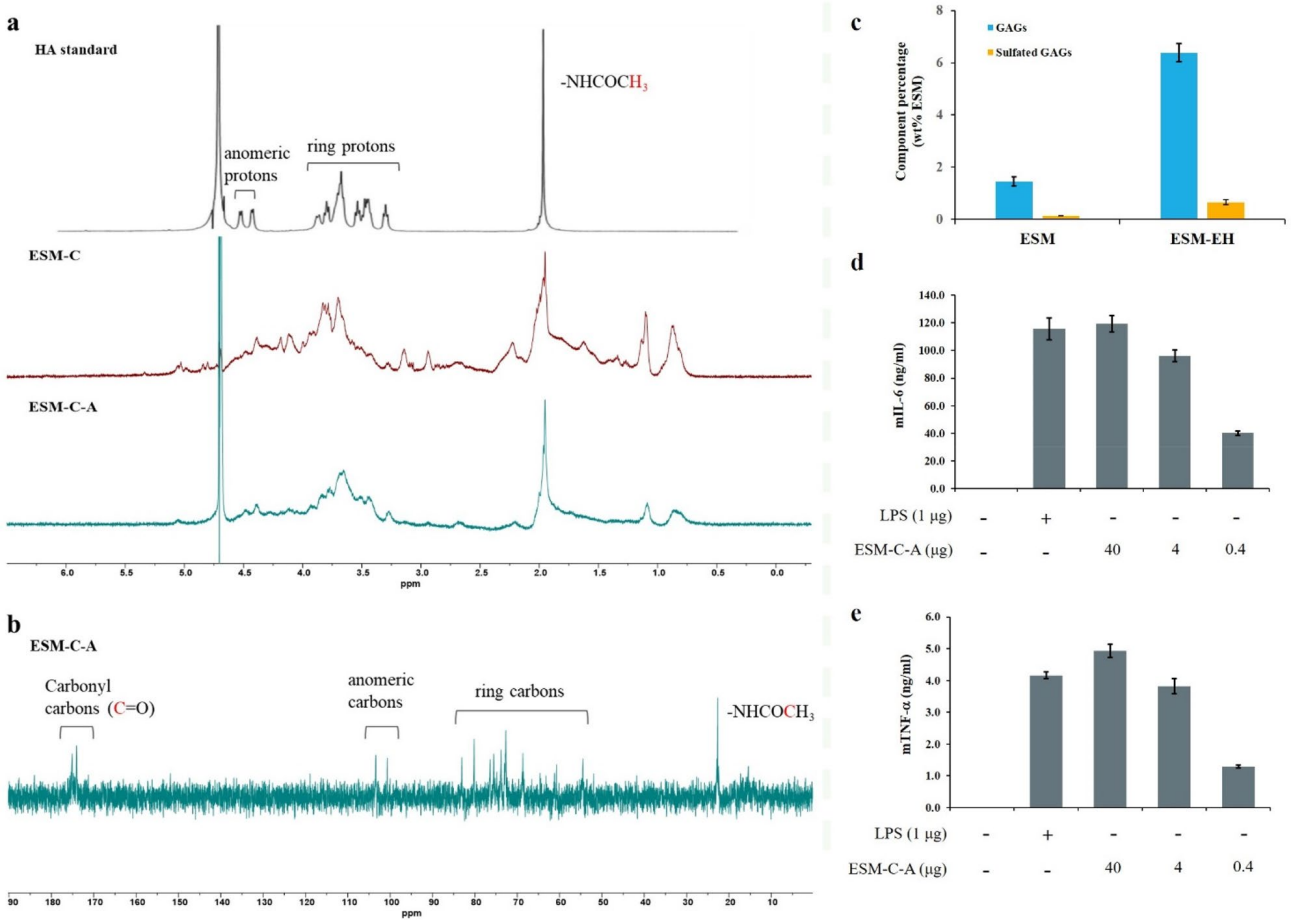
Analysis of ESM enzyme hydrolysate. (**a**) The ^1^H NMR spectrum of the HA standard^29^, the crude extract of polysaccharide (ESM-C), and the purified polysaccharides (ESM-C-A). (**b**) The ^13^C NMR spectrum of ESM-C-A. Characterization of ESM-C by NMR spectroscopy. The results showed that ESM-C was composed of HA. The spectra data was afforded on MestReNova (ver. 12.0.2) software. (**c**) The ratio of GAGs and sulfated GAGs in ESM enzyme hydrolysate. ESM refers to the control without treating with keratinase and Na_2_SO_3_ while ESM-EH refers to ESMP treated with keratinase and Na_2_SO_3_. The production of (**d**) IL-6 and (**e**) TNF-α was detected by the ELISA kit and indicates the immunomodulatory activity of ESM-C-A. Lipopolysaccharide (LPS) was used as a positive control to induce the inflammatory response from mouse J774A.1 macrophages. All the data are displayed as values averaged from at least three independent experiments.

According to a previous study, GAG is one of the valuable components in ESM, and it was precipitated from ESM-EH in this study for further characterization. The specific functional groups of ESM-EH were identified based on Fourier-transform infrared spectroscopy (FT-IR), which displayed similar spectra to hyaluronic acid (HA) (Supplementary Fig. S2). The high wavenumber region spectra showed an obvious 3340 cm^-1^ peak representing the signal of the O–H or NH group. At 1650 cm^-1^ (C=O), 1530 cm^-1^ (CN Stretching/NH bending mode), and 1203 cm^-1^ (CN stretching/NH bending mode), the peaks can correspond to the structure of amide I, amide II, and amide III, respectively^40^. The 1414 cm^-1^ peak represents the COO^-^ signal, which corresponds to the carboxyl group on HA. The peak at 636 cm^-1^ indicates the presence of CH bonds^41^. The crude polysaccharide precipitated from ESM-EH (ESM-C) was derivatized and its monosaccharide composition was determined by GC–MS (Fig. S3). According to the characteristic signals from different monosaccharides displayed at different residence times, the identifiable signals are roughly divided into 173 and 204 m/z, respectively^42^. The residence time from the GC–MS chromatograms of ESM-C at 173 and 204 m/z were 14–18 min and 19–21 min, respectively. Compared to the characteristic signals of monosaccharide standards at 204 m/z, the chromatograms revealed that ESM-C is composed of glucose, galactose, and mannose (Fig. S3b). The GC–MS chromatograms of ESM-C at 173 m/z were conserved to the peak retention time of HA, glucosamine, N-acetylglucosamine, galactosamine, and N-acetylgalactosamine standards (Fig. S3c). Among them, the chromatogram of ESM-C was highly consistent with the spectrum of HA (peaks 1, 4, and 5) and N-acetylgalactosamine (peaks 2, 3, and 4) in Supplementary Fig. S3c. To verify whether the component of ESM-C contained HA, the ^1^H NMR spectrum of ESM-C, and an isolated fraction of ESM-C obtained by size exclusion HW-65F chromatography (ESM-C-A), were compared to that of the HA standard^29^, which possessed three main regions corresponding to different protons (Fig. 2a). First, the signal representing N-acetyl (-NHCOCH_3_) can be found at the chemical shift of δ (ppm) 1.95; second, the ring protons accompanied by sugar are between δ (ppm) 3.0–4.0; and third, two of the anomeric protons are δ (ppm) 4.45 and 4.55, respectively. The ^13^C NMR spectrum of ESM-C-A (Fig. 2b) showed two obvious signals near δ (ppm) 175, which are carbonyl (C = O) signals. One is the carboxylic acid (COOH) on HA, and the other is N-acetyl (-NHCOCH_3_). There are two signals between δ (ppm) 100–105, which are the signal of anomeric carbons (the 1st carbon position on sugar). The δ (ppm) 50–85 are carbon signals on the sugar ring. Among them, the two signals of δ (ppm) 80–85 may be the carbon signals of the sugar linkage due to their higher chemical shift^43^. In summary, according to the information provided by these NMR spectra and compared with the standard, the main components of the ESM-C-A sample are disaccharides that are highly consistent with HA. Moreover, the average molecular weight of GAGs in ESM-EH was determined by using high-pressure size exclusion chromatography (HPSEC) combined with a Refractive Index detector (Fig. S5a). The results showed two obvious peaks, the residence time of which is 6.395 min and 7.514 min, corresponding to an average molecular weight range of 1300–1700 kDa.

### Determination of GAGs and sulfated GAGs from ESM-EH

Both GAGs and sulfated GAGs are considered as nutritional supplements, which thus possess economic value if they can be recycled from ESM. The GAGs and sulfated GAGs were quantified based on carbazole and DMMB, respectively, which were described in materials and methods. In this study, the oeMtaker-mediated ESM-EH was harvested and its GAGs and sulfated GAGs were quantified (Fig. 2c). The data showed that each gram of ESM contained 6.4±0.3% GAGs and 0.7±0.1% sulfated GAGs, which are higher than those in other studies^16,44^. Previous papers reported that 4.0–4.5% GAGs could be harvested from each gram of ESM by using trypsin digestion at 37 ℃ for 5 h^44^. Another published condition lead to 5% GAGs per gram of ESM by treating with pepsin at 40 ℃ for 5 h^16^ (Table 3). In this study, we provided a better enzymatic system to hydrolyze ESM and obtain more productive GAGs and sulfated GAGs for further application.

**Table 3:** The glycosaminoglycans content in the solubilized ESM by difference proteases.

Reference	This paper	Eva Ürgeová (2016)^44^	Eva Ürgeová (2016)^44^	Arsal Cocuľová (2018)^16^	Tanaka, S.-I. (2013)^15^	Liang, H. (2011)^45^
**Enzyme**	oeMtaker-mediated	Trypsin	Papain	Pepsin	Proteinase K	Trypsin and Papain
**Condition**	55 °C, 3 h	37 °C, 5 h	60 °C, 5 h	40 °C, 5 h	50 °C, 12 h	50 °C, 6 h
**GAGs ratio (wt% ESM)**	6.4%^a^	4.5%^a^	3.9%^a^	5%^a^	0.11%^b^	1.2%^a^

^a^Quantification is measured by carbazole assay.

^b^Quantification of the data is a commercially available hyaluronic acid measurement kit (Seikagaku Corporation).

### Polysaccharides isolated from ESM-C-A possess immunomodulatory activity

To determine if the polysaccharides harvested from ESM-EH are immunoactive, mouse J774A.1 macrophage cells were treated with ESM-C-A. The formation of interleukin-6 (IL-6) and tumor necrosis factor α (TNF-α) were dependent on the dose of ESM-C-A treatments (Fig. 2d, e). Polymyxin B (5 μg/ml) was used to confirm LPS contamination. As shown in Figure S6, polymyxin B treatment strongly inhibited LPS induced IL-6 and TNF-α production. As the same time, it was only slightly reduced using ESM-C-A, indicating the absence of endotoxin in the sample. High-molecular-weight HA is generally not thought to promote inflammatory responses in macrophages^46^, which is consistent with our results (Fig. S6). We speculate that the immunoactivity may arise from minor substances in ESM-C-A, especially chondroitin 4-sulfate, which has been reported to have pro-inflammatory activity^47^.

### Microorganism-based ESM hydrolysis system with *M. taiwanensis* WR-220

**Figure 3:**
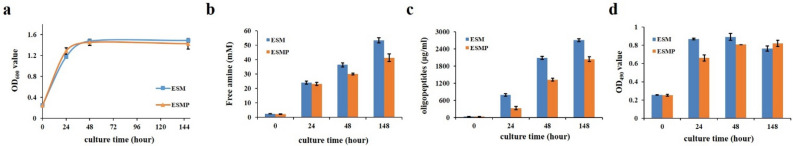
Microorganism-based ESM hydrolysis system. (**a**) The growth curves (OD 600) of *M. taiwanensis* WR-220 when using ESM or ESMP as sole nutrients for growth. (**b**) Concentration of free amines, (**c**) Concentration of oligopeptides and (**d**) Relative concentration of total sugar (OD 490) in ESM or ESMP hydrolysates during 148 h incubation with *M. taiwanensis* WR-220.

Based on the above evidence, we suggest that the oeMtaker-mediated ESM hydrolysis system is superior to those previously described. To scale up the reaction for further industrial application, a microorganism-based ESM hydrolysis system was established. In this system, ESM or ESMP was added as the sole nutrient source for *M. taiwanensis* WR-220. After 48 h of cultivation, red pigments could be observed, which indicated the growth of *M. taiwanensis* WR-220. After 148 h of cultivation, both ESM and ESMP were decomposed as homogeneous pulp in solution (Fig. S7). According to the growth curve, the bacterial population reached a plateau after 48 h in culture. There is no difference in whether using ESM or ESMP as a major nutrient source (Fig. 3a). Theoretically, the number of released free amino acids and oligopeptides treated with Mtaker could be considered a factor to monitor ESM decomposition. On the other hand, these species may be used by *M. taiwanensis* WR-220 for growth during incubation. The ninhydrin colorimetric assay and Bradford assay results showed the remaining amounts of free amine and oligopeptides in ESM or ESMP hydrolysates continued to increase over time. The remaining free amine and oligopeptides released from ESM became slightly higher than ESMP when the incubation time extended from 24 to 144 h (Fig. 3b, c). We reasoned that ESMP is more suitable as a nutrition source than ESM due to the larger surface area and that the enzyme can more easily access the substrate. Based on results of carbohydrate concentration in the phenol–sulfuric acid method (OD490), the carbohydrate content in ESM and ESMP hydrolysates was increased by about four times after being cultured with the bacteria (Fig. 3d). In addition, after 24, 48, and 144 h of incubation, the remaining ESM in the ESM-EH were collected by centrifuged, washed with water, and dried at 55 °C. The calculated weight decomposition percentage is about 17%, 27%, and 40%, respectively. It is important for product application development and safety that *M. taiwanensisis* does not produce lipopolysaccharide in their outer membrane even though the organism is a gram-negative bacteria^48^.

In a subsequent pre-pilot test of the microorganism-based ESM hydrolysis system, the reaction volume was scaled-up to 3.0 L and contained 90 g of eggshell membranes as the *M. taiwanensis* WR-220 major nutrient source. This 3 L culture was incubated in a fermentation tank to monitor its real-time temperature, pH, and rotating speed. During three days of fermentation, the optical cell density reached 1.4 at 600 nm, similar to the 0.1 L culture system. The maximal released free amine from the 3 L fermentation system was 24 mM while it could reach 36 mM when the culture was in 0.1 L system after 48 h incubation. We also quantified GAGs (0.9%/g ESM) and sulfated GAGs (0.1%/g ESM) from the 3 L fermentation system after three-day incubation. Compared to the oeMtaker-mediated ESM hydrolysis system, the scale-up of the microorganism-mediated ESM hydrolysis system to 3 L resulted in fewer GAGs and sulfated GAGs that could be harvested (Table S2). We hypothesize that this may be due to the culture condition of the microorganism-mediated ESM hydrolysis system, which does not include sodium sulfite as a reducing agent.

## Discussion

In summary, the best keratinase hydrolyzing ESM condition we have identified involves adding 0.650 μM keratinase and 50 mM sodium sulfite, then incubating at 55 °C for 3 h. This results in a weight ratio of oeMtaker:sodium sulfite:ESM of 1:120:600. The procedures of ESM hydrolysis by strong acid or strong alkali solutions are usually coupled with high-temperature incubation under high-pressure conditions. Through both chemical and physical treatments, ESM could be decomposed into smaller constituents. However, these reactions need further neutralization to make its product usable. Lots of the bioactive ingredients may be destroyed by these violent processes, resulting in low yields of recycled nutrients for further application. In the composition analysis of the ESM-EH, the ratio of serine, methionine, and essential amino acids is higher than that produced by acid hydrolysis. This finding demonstrates that using keratinase to hydrolyze eggshell membranes is milder than acid hydrolysis. The amino acids contained in the ESM-EH are suitable for use as nutritional supplements and animal feed. Among the enzymatically hydrolyzed ESM published so far, the glycosaminoglycan ratio (wt%) of ESM treated with pepsin at 40 °C for 5 h was 5%^16^. The treatment with trypsin and papain at 50 °C for 6 h yielded a GAGs ratio of 1.2% ^45^ while the ESM incubated with trypsin at 37 °C for 5 h could turn out 4.5% GAGs^44^. Another published condition made 0.11% GAGs out of ESM by treating with proteinase K at 50 ℃ for 12 h^15^ (Table 3). In this study, we discovered the best enzymatically ESM hydrolysis condition that a carbohydrate ratio 6.4% was achieved by incubating ESM with oeMtaker and sodium sulfite at 55 °C for 3 h. Proteomics data confirmed that the ESM-EH is rich in collagen peptides with molecular weight of about 963–2259 Da. Among them, type X collagen is the major species. Collagen peptides with smaller molecular weights are better absorbed and used by the body than larger fragments. The enzyme hydrolysate of ESM has also been successfully extracted as a crude extract (ESM-C). Analysis by GC–MS, FT-IR spectroscopy and NMR spectroscopy confirmed that this fraction is carbohydrate in nature and appears to contain which HA, N-acetyl-D-galactosamine, D-galactose, and D-mannose. Furthermore, a major component of ESM-C (ESM-C-A), obtained by size exclusion chromatography, has a molecular weight range of 1300 to 1700 kDa. Based on the carbazole and DMMB results, the ratio of unsulfated GAGs is higher than sulfated GAGs. The ^1^H and ^13^C NMR spectrum are used to infer that ESM-C-A mainly contains carboxylic acid groups (COOH), sugar rings, and acetoxy groups (OCH_3_), which is indicative of high molecular weight HA. This fraction contains an immunologically active ingredient, which may come from small amounts of carbohydrates other than HA. It is likely chondroitin 4-sulfate, which has been reported to possess pro-inflammatory activity. This strategy is an advanced technology for the biological reuse of ESM and the digestive product of ESM provides more economic benefits.

## Supplementary Information

Supplementary Information.

## Data Availability

All data generated or analyzed during this study are included in this published article and its supplementary information files.
