# Supplementary material for: High-efficiency decomposition of eggshell membrane by a keratinase from Meiothermus taiwanensis
Source: Sci Rep. 2022 Aug 29;12:14684. doi: 10.1038/s41598-022-18474-4 (PMC9424195; doi:10.1038/s41598-022-18474-4)
Supplement: Supplementary file 1 — Supplementary Information. [file 41598_2022_18474_MOESM1_ESM.pdf]

Supplemental information

High-efficiency decomposition of eggshell membrane by a keratinase from *Meiothermus taiwanensis*

Ya-Chu Lien[1], Shu-Jung Lai[2, 3], Chai-Yi Lin[1], Ken-Pei Wong[4, +], Matt S. Chang[4, +], and Shih-Hsiung Wu[1, *]

1 Institute of Biological Chemistry, Academia Sinica, No. 128, Academia Road, Section 2, Nankang, Taipei 11529, Taiwan

2 Graduate Institute of Biomedical Sciences, China Medical University, No.91, Hsueh-Shih Road, Taichung, 40402, Taiwan

3 Research Center for Cancer Biology, China Medical University, No.91, Hsueh-Shih Road, Taichung, 40402, Taiwan

4 I-MEI FOODS Company Limited, 1 F., No. 31, Sec. 2, Yanping N. Rd., Datong Dist., Taipei City 10346, Taiwan

* corresponding author
Shih-Hsiung Wu
Institute of Biological Chemistry, Academia Sinica, No. 128, Academia Road, Section 2, Nankang, Taipei 11529, Taiwan
Phone: +886-2-27855696 ext.7101
* E-mail: shwu@gate.sinica.edu.tw
[+]these authors contributed equally to this work

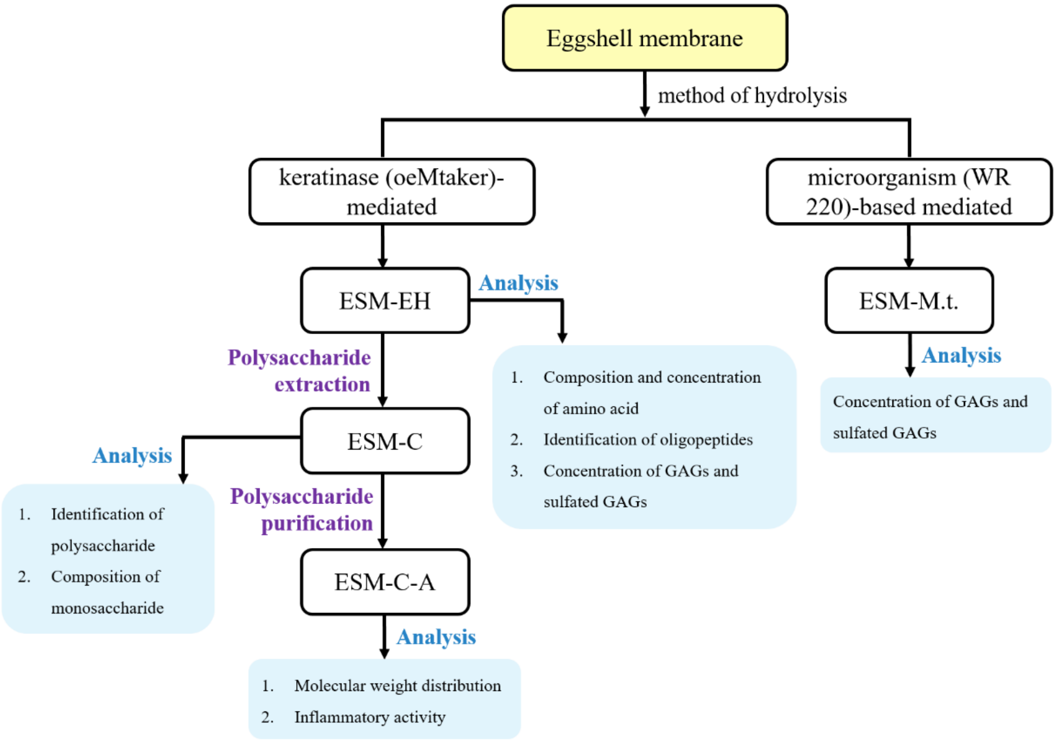

Figure S1. Flowchart of decomposition of ESM.

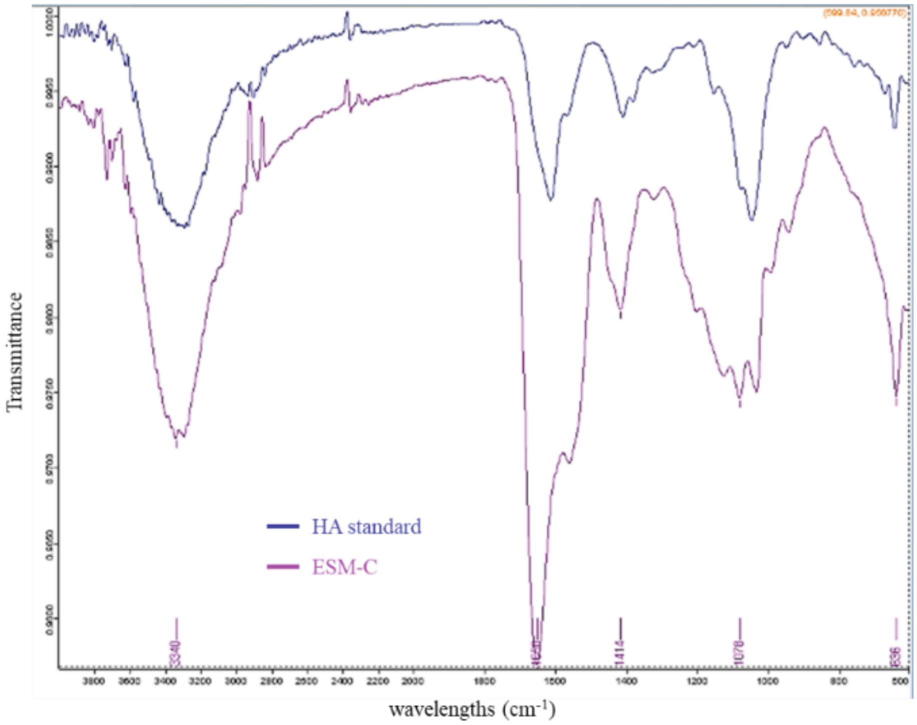

Figure S2. Fourier transform infrared (FT-IR) spectra of HA standard (blue line) and ESM-C (purple line). The spectra was afforded on Bruker TENSOR 27 FT-IR spectrometer and OPUS data collection program v6.5 (https://www.bruker.com/en.html).

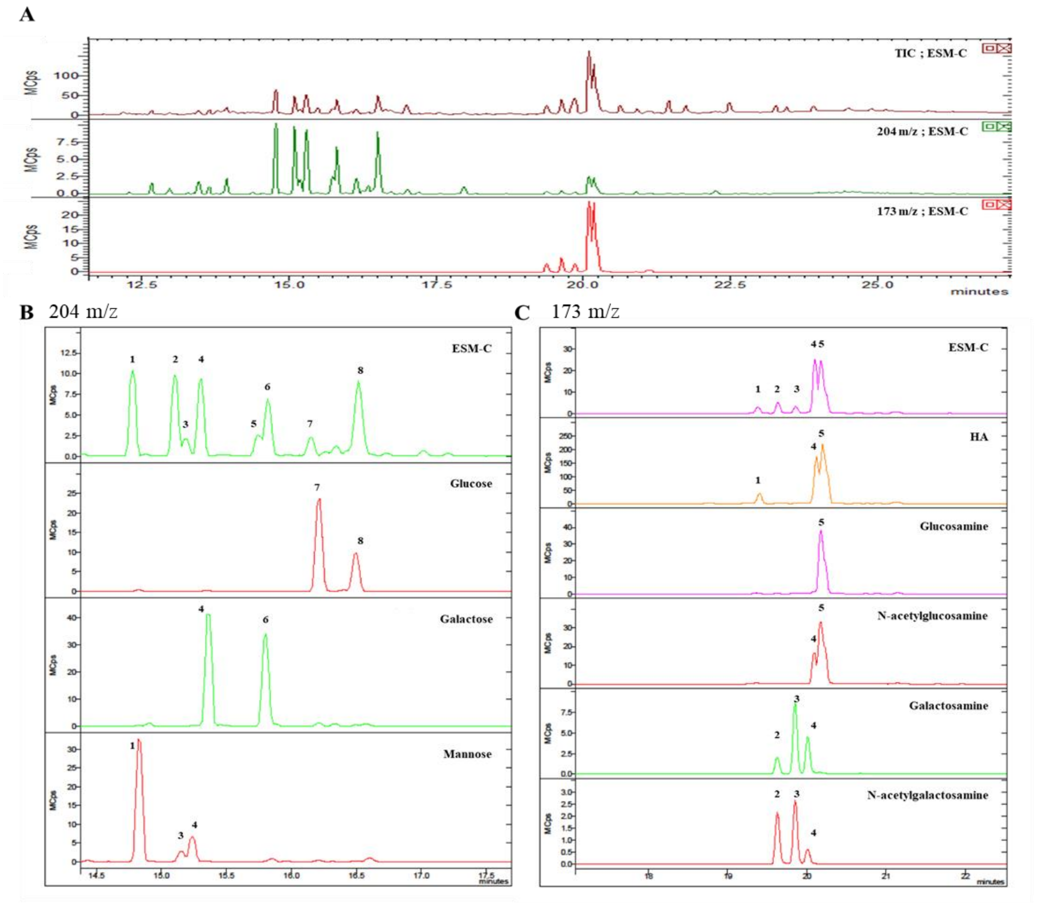

Figure S3. GC-MS chromatograms of ESM-C in comparison to monosaccharide standards. (A) Total ion chromatogram (TIC), 204 m/z extracted ion chromatogram (EIC), and 173 m/z EIC of ESM-C. (B) 204 m/z EIC of ESM-C and standards (C) 173 m/z EIC of ESM-C and standards. The profile was afforded on Bruker SCION SQ GC-MS and SCION MS Workstation v8.2.1 (https://scioninstruments.com).

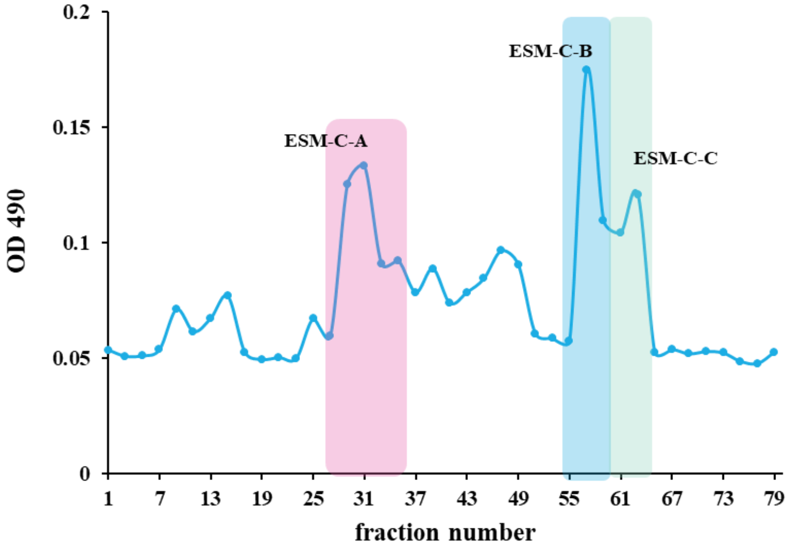

Figure S4. Size exclusion HW-65F chromatogram of the crude extract of polysaccharide (ESM-C). Fractions collected were monitored by the phenol-sulfuric acid reaction[1] (absorbance at 490 nm). The ESM-C-A (11 mg), ESM-C-B (< 1 mg), and ESM-C-C (< 1 mg) samples were collected from fraction numbers 27-35, 55-60, and 61-65, respectively.

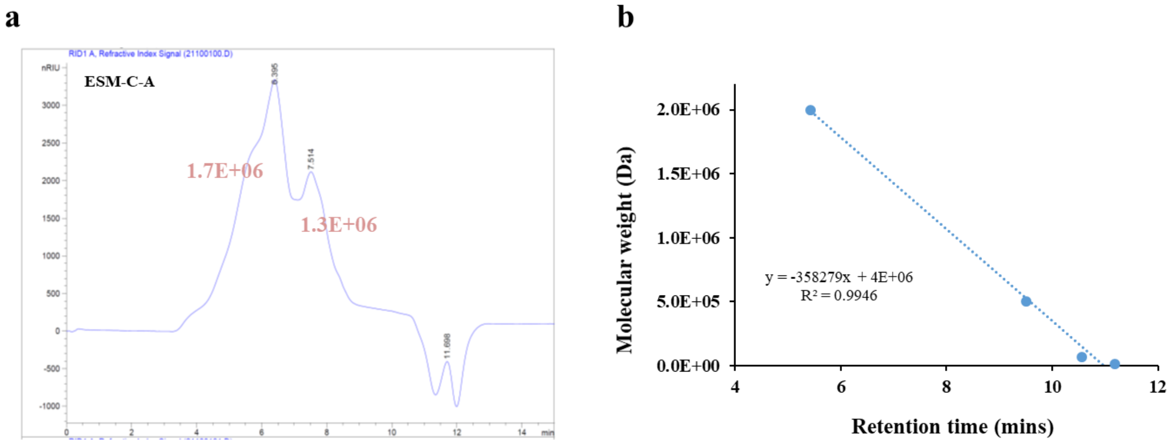

Figure S5. The HPSEC spectra (a) showing the average molecular weight of ESM-C-A, and the standard curve (b) using different dextran (10 kDa, 70 kDa, 500 kDa and 2000 kDa) sample standards.

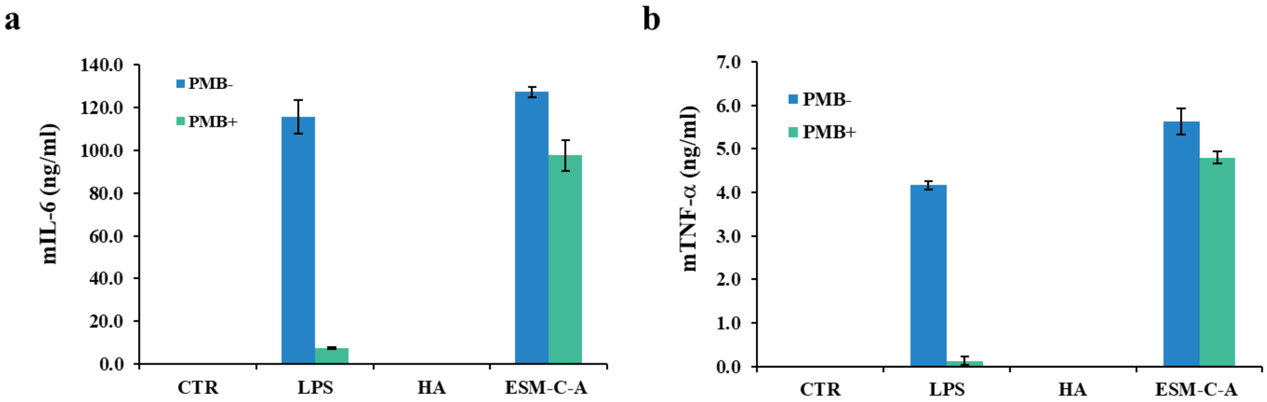

Figure S6. PMB assay of ESM-C-A. The production of (a) IL-6 and (b) TNF-α was detected by the ELISA kit. ESM-C-A was demonstrated to be endotoxin-free. HA is mean sodium hyaluronate with a molecular weight of 1500~1800 kDa, purchased from Sigma-Aldrich.

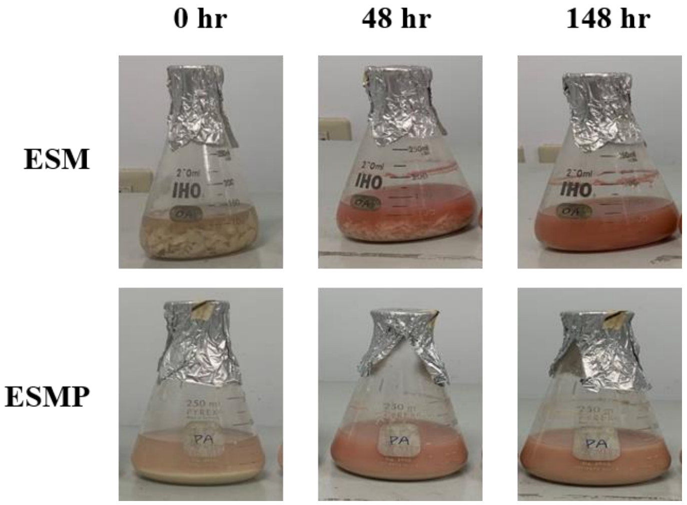

Figure S7. Microorganism-based ESMs hydrolysis system. The observed decomposition ability of ESM and ESMP during 148 h incubation with *M. taiwanensis* WR-220 were compared. The red pigment released from *M. taiwanensis* WR-220 during incubation could be one of the factors that correlate to its growth.

Table S1. Identified other peptides in ESM hydrolysate by LC-MS/MS analysis

**Proteins ID**	**Peptides sequence**	**MS/MS m/z**	**Mass**	**Score**
Lysyl oxidase homolog 2	PYLP*GYPEPPPQVY	816.8997	1631.7872	173
	VGGRNPYEGR	552.7802	1103.5472	135
	RNPYEGRVE	560.2796	1118.5469	119
	PYLPGYPEPPPQVY	808.9014	1615.7923	118
	GQSDFRPK	467.7399	933.4668	115
	PYLPGYPEPPPQ	677.8363	1353.6605	115
	GGRNPYEGRVE	617.3018	1232.5898	108
	DVPPGDYLF	511.7453	1021.4757	107
	PYLP*GYPEPPPQ	685.8340	1369.6554	107
	NNGQSDFRPK	581.7824	1161.5527	104
	PYLP*GYPEPPPQ	685.8346	1369.6554	89
Vitellogenin-2	GKM(ox)TPPLTGDF	590.2881	1178.5642	161
	MTPPLTGDF	489.7332	977.4528	121
Ovalbumin	YRGGLEPINFQ	647.3323	1292.6513	126
	DKLPGFGDSIEAQ	688.8371	1375.6620	122
	RGGLEPINFQ	565.8002	1129.5880	118
Phosphatase and actin regulator 4	EGKEAAWP*	452.7057	902.4134	125
Vitellogenin-1	LETKPGLPIL	540.8355	1079.6591	119
Cytoplasmic envelopment protein 1	KTIGIP*PP*L	484.2961	966.5750	112
Ovocalyxin-32	ERLPWPQVPGVM	704.8730	1407.7333	98

P*: hydroxyproline

The data was afforded on MaxQuant (ver. 1.6.6.0) and *Gallus gallus* total protein database which was downloaded on 11th Jun 2021 from Uniprot (https://www.uniprot.org).

Table S2. Comparison of the decomposition conditions between oeMtaker-mediated and microorganism-mediated ESM hydrolysis system

Name	Methods	Incubation time	Decomposition percentage (wt% ESM)	Released free-amine (mM)	GAGs (wt% ESM)	Sulfated GAGs (wt% ESM)
**ESM-EH**	oeMtaker-mediated	3 hrs	81.5±2.5	24.6±1.1	6.4±0.3	0.7±0.1
**ESM-M.t.**	microorganism mediated*	72 hrs	ND**	24.1±0.5	0.9±0.4	0.1±0.0

* without sodium sulfite, ** no detection.

Supporting Materials and Methods

Phenol-sulfuric acid method. The isolated carbohydrate fractions were detected by the phenol-sulfuric acid method.[1] To 100 µL of each eluted fraction was added 500 µL of concentrated sulfuric acid and 100 µL of 5% phenol solution, and then shaken to mix well at room temperature. The reaction is exothermic. After cooling to room temperature, the absorbance was measured at a wavelength of 490 nm.

Fourier transform infrared (FTIR) spectrum of ESM-C. The FTIR spectrum[2] of the ESM-C sample was obtained and analyzed by using Bruker Tensor-27 IR from 4000 to 400 cm^{-1}.

References

1. Masuko, T. *et al.* Carbohydrate analysis by a phenol-sulfuric acid method in microplate format. *Anal Biochem* **339**, 69–72, DOI: https://doi.org/10.1016/j.ab.2004.12.001 (2005).
2. Zhao, Y.-H. & Chi, Y.-J. Characterization of Collagen from Eggshell Membrane. *Biotechnology* **8**, 254–258, DOI: https://doi.org/10.3923/biotech.2009.254.258 (2009).
